# Arthroscopically Assisted Coracoclavicular (CC) Stabilization Using a Suture Button Device for Lateral Clavicle Fractures with CC Ligament Injury

**DOI:** 10.3390/jcm13061773

**Published:** 2024-03-20

**Authors:** Yoshimasa Saigo, Daichi Morikawa, Yoshiaki Itoigawa, Hirohisa Uehara, Takayuki Kawasaki, Takefumi Kaketa, Kenta Shibuya, Hironori Tsurukami, Fumitoshi Hatae, Yasutaka Yoshimura, Kazuki Yoshida, Muneaki Ishijima

**Affiliations:** 1 Department of Orthopaedics, Juntendo University Faculty of Medicine, Tokyo 113-0034, Japan; y.saigo.ux@juntendo.ac.jp (Y.S.); yitoiga@juntendo.ac.jp (Y.I.); hi-uehara@juntendo.ac.jp (H.U.); k-saki@juntendo.ac.jp (T.K.); t-kaketa@juntendo.ac.jp (T.K.); ke-shibuya@juntendo.ac.jp (K.S.); h-tsurukami@juntendo.ac.jp (H.T.); f-hatae@juntendo.ac.jp (F.H.); y-yoshimura@juntendo.ac.jp (Y.Y.); k.yoshida.cz@juntendo.ac.jp (K.Y.); ishijima@juntendo.ac.jp (M.I.); 2 Department of Orthopaedic Surgery, Juntendo University Urayasu Hospital, Urayasu 279-0021, Japan

**Keywords:** lateral clavicle fracture, coracoclavicular ligament, modified Neer classification, arthroscopic surgery

## Abstract

**Background:** Lateral clavicle fractures represent approximately 10–15% of all clavicle fractures. However, controversy exists regarding the optimal surgical treatment because of instability associated with the coracoclavicular (CC) ligament injury and a small lateral fragment. The purpose of this study was to evaluate the radiological and clinical outcomes of arthroscopically assisted CC stabilization using a suture button device for lateral clavicle fractures accompanied by CC ligament injury. **Methods:** A retrospective observational study involved six patients with modified Neer type IIB fractures, which were treated with the technique and followed for 12 months. Postoperative range of motion (ROM) and X-rays were evaluated every 3 months. Shoulder functional scores (University of California Los Angeles score, Japanese Orthopedics Association score) and visual analog scale (VAS) scores for pain (at rest, at night, and during motion) and for satisfaction were analyzed 12 months after surgery. **Results:** Early phase ROM recovery and excellent outcomes were achieved. All patients achieved bone union. Slight superior clavicle displacement and bone hole dilation occurred with no critical complications. **Conclusions:** Arthroscopically assisted CC stabilization with a suture button device for unstable lateral clavicle fractures can produce satisfactory radiological and clinical results.

## 1. Introduction

Clavicle fractures are very common, with an incidence of 30 per 100,000 [1]. These fractures represent 2.6–4% of all fractures [2,3]. Fractures of the clavicle shaft have the highest incidence among clavicle fractures and account for 69% of all clavicle fractures. The classical injury mechanisms are simple falls on the shoulders (31%), followed by road traffic accidents (27%) and sports (23%) [1]. Lateral clavicle fractures represent approximately 10–15% of all clavicle fractures [4] and 50% of all ununited clavicle fractures [5]. An analysis of the frequency of lateral clavicle fractures using age and sex has revealed that these fractures occur most often in men between 30 and 50 years of age and, secondarily, in individuals (both men and women) over 70 years of age [6]. These fractures have a high nonunion rate (33%) after nonsurgical treatment [5]. Nonunions and malunions can result in persistent pain, restricted range of motion (ROM), and loss of strength. In the largest available series of nonoperatively managed displaced lateral clavicle fractures, 14% of patients eventually required surgery [7], and reconstruction of an established nonunion can be technically challenging [8]. Therefore, surgical management is commonly offered for displaced lateral clavicle fractures, and multiple surgical procedures are available. The Neer classification system has been a common classification system for lateral clavicle fractures. This classification is based on the fracture location in relation to the coracoclavicular (CC) ligament on simple anteroposterior radiographs and its involvement [9,10]. In 1990, Craig introduced a modified version of the Neer classification, which is more detailed and helpful in determining treatment and prognosis [11]. Currently, lateral clavicle fractures are commonly classified using the Neer classification, which Craig modified [12,13,14,15,16,17,18,19]. The modified Neer classification divides lateral clavicle fractures into types I–V. Type I fractures occur lateral to the CC ligament with minimal displacement and no involvement of the acromioclavicular (AC) joint. Type III fractures are similar to type I (i.e., these also lie lateral to the CC ligament) but have intra-articular extension without AC joint injury [20]. Type I and Type III fractures with an intact CC ligament and AC ligament are stable and can be treated conservatively [21]. Type IV fractures involve disruption of the periosteal sleeve, and the medial fragment gets displaced upwards. Type IV fractures are more common in children than in adults [20]. Type V fractures are similar to Type II (i.e., involving a small inferior fragment attached to the CC ligament) and are comminuted [20]. Type II fractures are considered unstable with a higher risk of nonunion or malunion and are considered for surgical management, most often among the fracture types [20]. Type II fractures are further subcategorized into types IIA and IIB. In type IIA fractures, the CC ligament remains intact, whereas in type IIB injuries, the CC ligament is partially or completely detached [22]. The optimal treatment of type IIB lateral clavicle fractures is especially controversial because of the instability associated with CC ligament injury and the difficulty of achieving accurate fixation with a small lateral fragment [11].

Numerous surgical techniques for type IIB lateral clavicle fractures, including K-wire fixation, tension band wiring (TBW), CC screw fixation, hook plate fixation, anatomical locking plate fixation, and CC ligament reconstruction, have been reported [23]. However, there is no gold standard for surgical treatment of this type of fracture [22] because there are no available randomized control trials, and most studies were reports of small case series. The largest amount of available evidence comes from a few studies that compared only hook plate fixation and locking plate fixation [24,25]. A review found no superiority for any techniques based on functional outcomes and union rates [26]. K-wire fixation and TBW have been used for a long time but have decreased in popularity because of complications such as K-wire migration, implant failure, pseudoarthrosis, and infection [6]. Hook plate fixation clearly has a high complication rate among surgical techniques for type IIB lateral clavicle fractures, and these plates are always removed as soon as possible, depending on fracture consolidation. Subacromial impingement, rotator cuff lesions, acromial fractures, implant failure, and especially implant-related irritation are reported in 40–70% of patients [6,24,25].

CC stabilization using a suture button device is a relatively new treatment for type IIB lateral clavicle fractures. This procedure is especially appropriate for fresh modified Neer Type IIB fractures. Previous risk analysis showed that a lateral clavicular fragment larger than 3 cm and delayed surgery were risk factors for non-union [27]. A biomechanical study revealed that CC stabilization using a CC button and suture tapes produced greater stability for superior loading compared with a distal third locking plate in type IIB lateral clavicle fractures [28]. Other advantages of CC stabilization for type IIB lateral clavicle fractures were the lower necessity for implant removal and the fact that there was no need for AC joint fixation compared with prior hook plate fixation [29]. Several studies have reported satisfactory clinical and radiological results after CC stabilization using a suture button device for type IIB lateral clavicle fractures [13,14,30,31,32]. Arthroscopic techniques were reported primarily to manage AC joint problems successfully, and some authors have proposed arthroscopic surgery or arthroscopically assisted surgery for the treatment of lateral clavicle fractures [12,13,14]. Most studies performed CC fixation with arthroscopic reduction of displaced fractures to obtain several key advantages: minimal invasiveness, early rehabilitation, less postoperative pain and wound problems, and ability to address concurrent shoulder joint problems, such as impingement, rotator cuff tear, or superior labrum anterior and posterior lesions [6,33]. Considering these advantages, mini-open reduction and arthroscopically assisted CC stabilization using a suture button device were adopted in our hospital.

Since 2021, we have performed mini-open reduction and arthroscopically assisted CC stabilization using an AC Dog Bone Button device (Arthrex, Naples, FL, USA) as a suspension system. The system showed higher stability for superior loading and required minimal bone tunnels (2.4 mm) for the coracoid and clavicle compared with other systems, thereby reducing the risk of bone tunnel-related fractures in the coracoid and clavicle [28]. The purpose of the present study was to evaluate the clinical and radiological outcomes of mini-open reduction and arthroscopically assisted CC stabilization using the AC Dog Bone Button device for type IIB lateral clavicle fractures accompanied by CC ligament injury.

## 2. Materials and Methods

### 2.1. Patients

From January 2021 to February 2022, six consecutive patients with modified Neer type IIB lateral clavicle fractures and CC ligament injury underwent mini-open reduction and arthroscopically assisted CC stabilization using the AC Dog Bone Button device (Arthrex, Naples, FL, USA) by a senior surgeon (D.M.). All patients were classified via preoperative X-ray and computed tomography (CT) examinations and followed up for a minimum of 12 months.

### 2.2. Surgical Technique

#### 2.2.1. Presurgical Measurement

Bilateral X-rays and CT images were used for presurgical planning. The position of the smallest distance between the clavicle and coracoid process was measured for bone tunnel creation in the clavicle and coracoid in the uninjured side CT images. The amount of superior and posterior dislocation was measured to estimate the reduction landmark on the injured side.

#### 2.2.2. Installation and Anesthesia

All surgical interventions were conducted under general anesthesia with the patient in the beach chair position. An image intensifier and an arthroscopic monitor were positioned opposite the operated shoulder. A three-dimensional support arm was used to stabilize the patient’s arm on the injured side.

#### 2.2.3. Mini-Open Reduction

A 5 cm incision (4 cm proximal and 1 cm distal to the fracture line) along the bone axis was created on the clavicle and AC joint. The delta-trapezius fascia was incised to expose the clavicle periosteum. The anterior and posterior limits of the fracture were then exposed with periosteum trimming. Scar tissue around the fracture was removed, and the AC joint was localized without invasion to identify the landmark for reduction. The fixation point, based on the presurgical measurement, was marked. Manual open reduction was performed, and the reduction was confirmed both directly and with intraoperative fluoroscopic imaging. A 1.8 mm K-wire, chosen on the basis of the bone size and degree of osteoporosis, was then inserted from the acromion to the clavicle for fixation. Accurate reduction was confirmed through intraoperative fluoroscopy.

#### 2.2.4. Arthroscopic Approaches

Two standard approaches were used. The posterior glenohumeral approach was used first, 2 cm below and 2 cm inside the posterolateral edge of the acromion. The optical standard at 30° was used, and the instrumental anterosuperior portal was established through the rotator interval using an outside-in technique.

#### 2.2.5. Arthroscopic Exploration and Exposure

Diagnostic shoulder arthroscopy was performed initially to explore the glenohumeral cavity and check for associated lesions. After opening the rotator interval and identifying the coracoid process, the anterolateral portal was created to allow easy access to the coracoid base using an outside-in technique. Using the scope from the posterior portal, the base of the coracoid process was progressively exposed using electrocoagulation [13]. During this step, a 70° arthroscope was used to clearly show the medial coracoid base at the guide insertion point.

#### 2.2.6. Fixation

A C-shaped ancillary drill guide was inserted through the anterolateral portal. A canula was then inserted, and to avoid soft tissue entrapment when the Dog Bone Button device was placed, the C-shaped guide was positioned under the center of the coracoid process. The other end of the guide was applied and placed on the presurgical marked point, which had been exposed when the reduction was performed [34]. A 2.4 mm cannulated drill was passed through the clavicle and coracoid process under radioscopic guidance. Of note, when drilling the superior coracoid, it is easy for the drill bit to slip because of the curved shape of the bone. Therefore, cautious and gentle high-speed drilling is needed. After the removal of the ancillary instrument, leaving the pin, a lasso was passed through the cannulated drill and retrieved through the anterolateral portal. The drill was then removed. Two FiberTapes (Arthrex, Naples, FL, USA) and the Dog Bone Button device were passed from the inferior side of the coracoid process using a grasper to avoid tangling the FiberTapes. We then checked that the Dog Bone Button device closely contacted the coracoid base, using both arthroscopy and intraoperative fluoroscopy. A superficial Dog Bone Button device was also set, and the dislocation was reduced manually by tightening the device’s proximal end. The image was checked while removing the fixation K-wire, and the FiberTapes were tightened four times to secure the system and confirm the fixation of the Dog Bone Button device. Finally, the incisions were closed, with fascial wrapping to cover the knots and the implants.

#### 2.2.7. Postoperative Care

The patients used an arm sling for 4 weeks. Two weeks after surgery, the patients started passive and active ROM exercises comprising anterior elevation (AE) and abduction within 90 degrees. Four weeks after surgery, the patients were allowed to perform passive and active shoulder ROM exercises with no limitation. All patients were seen at 1 month for the first radiological and clinical follow-up visit and every 3 months until 12 months after surgery.

#### 2.2.8. Evaluation Criteria and Follow-Up

Postoperative follow-up took place as consultations, with systematic ROM measurements (AE, external rotation (ER), and internal rotation) every 3 months. Shoulder functional scores (University of California Los Angeles (UCLA) score, Japanese Orthopedics Association (JOA) score), visual analog scale (VAS) scores for pain at rest, at night, and during motion, and VAS scores for satisfaction were analyzed 12 months after surgery.

**Figure 1:**
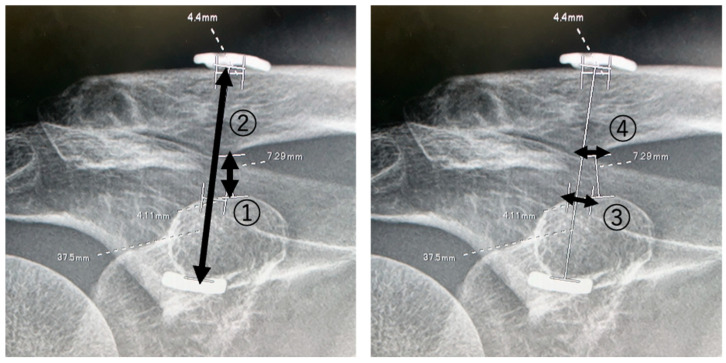
Measurements of the CC distance, Dog Bone distance, and bone holes on the superior coracoid and inferior clavicle. (1): CC distance; (2): Dog Bone distance; (3): superior coracoid bone hole; (4): inferior clavicle bone hole.

Standard anteroposterior and axial clavicle X-rays were taken systematically. Bone union, clavicular displacement, and bone hole sizes were assessed. Clavicular displacement was evaluated by measuring both the CC distance and the Dog Bone distance (Figure 1). Bone hole sizes were measured on the superficial side of the coracoid process and the inferior side of the clavicle in the anteroposterior position 1 week, 3, 6, 9, and 12 months after surgery (Figure 1).

### 2.3. Statistical Analysis

The differences between the follow-up visits were analyzed through the Friedman test. Bonferroni post hoc multiple comparisons were used to identify which pairwise comparisons reached statistical significance among the CC distance, Dog Bone distance, and bone hole sizes in the superior coracoid and inferior clavicle. An α level of 0.05 was considered significant for all analyses. Calculations were performed using IBM SPSS v.20 statistical software (IBM Corp., Armonk, NY, USA) on a personal computer.

## 3. Results

The patients’ characteristics and injury types are shown in Table 1. Three patients were injured in bicycle accidents, two patients were injured in falls, and the final patient was injured in a traffic accident. The patients comprised five men and one woman, and the mean age was 46.7 ± 12.2 years. The mean surgery time and amount of bleeding were 75.8 ± 8.2 min and 20.2 ± 10.8 mL, respectively.

### 3.1. Functional Outcomes

**Figure 2:**
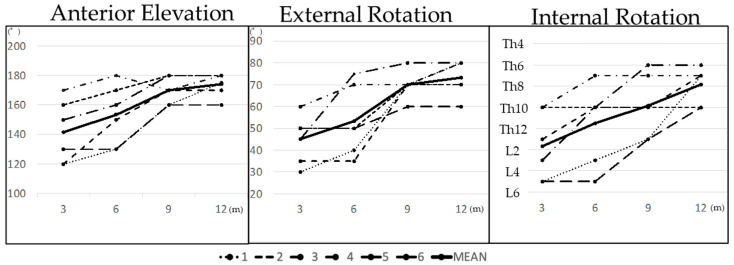
Postoperative ROMs.

The postoperative ROMs 3, 6, 9, and 12 months after surgery were 141.7 ± 19.5, 153.3 ± 18.9, 170.0 ± 8.2, and 174.2 ± 7.3 for AE; 45.0 ± 10.0, 53.3 ± 14.6, 70.0 ± 5.8, and 73.3 ± 7.5 for ER; and L2 ± 2.7, Th11 ± 3.4, Th10 ± 2.7, and Th8 ± 1.6 for internal rotation, respectively. AE and ER recovered almost completely between 6 and 9 months postoperatively; IR was increased gradually at these time points (Table 1 and Figure 2). Regarding the shoulder functional scores, the mean JOA and UCLA scores were 98.0 ± 3.1 and 34.3 ± 1.0, respectively, 12 months after surgery (Table 1). The mean VAS scores for pain 12 months after surgery were 0 at rest, 0 at night, and 0.3 ± 0.8 during motion (Table 1). The mean VAS score for patient satisfaction was 91.7 ± 5.2 12 months after surgery (Table 1).

### 3.2. Radiological Analysis

**Figure 3:**
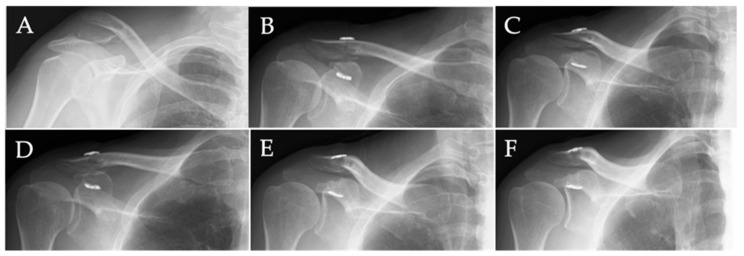
Representative X-rays before and after surgery. (**A**) Preoperative radiograph. (**B**) Postoperative radiograph 1 week after surgery. (**C**) Radiograph 3 months after surgery. (**D**) Radiograph 5 months after surgery showing complete bone union with subsidence of the clavicular button. (**E**) Radiograph 9 months after surgery showing no dislocation. (**F**) Radiograph 12 months after surgery showing no dislocation.

All six patients showed complete bone union, with two showing union at 3 months, two showing union at 5 months, and the remaining two showing union at 7 months. Representative X-rays before and after surgery are shown in Figure 3, showing that the patient achieved bone union at 5 months.

For both the CC distance and Dog Bone distance, there were no significant differences between all pairwise after surgery (Table 2). For the bone hole size in the superior coracoid, there were significant differences between measurements taken at 1 week and 9 and those at 12 months after surgery (*p* = 0.014 and *p* = 0.001, respectively) (Table 3). For the bone hole size in the inferior clavicle, there were significant differences between 1 week and 12 months after surgery (*p* = 0.001). There were no significant differences in the bone hole sizes in both the superior coracoid and inferior clavicle 3 months after surgery (Table 3).

There were no perioperative complications, including nonunion, implant failure, peri-implant fracture, deep infection, coracoid and acromial fractures, delayed union, implant problems, subacromial osteolysis, clavicular erosion, peri-anchor problems, loss of reduction, AC joint arthrosis, pain, and superficial infection.

## 4. Discussion

In the present study, we analyzed the clinical and radiological results of arthroscopically assisted CC stabilization using a suture button device for lateral clavicle fractures accompanied by CC ligament injury (type IIB fracture in the modified Neer classification). The major findings of the study were early phase recovery of active shoulder ROM (i.e., acceptable at 3 months), satisfactory clinical outcomes in two scoring systems, excellent bone union with slightly delayed healing, and a low complication rate.

For the recovery of active shoulder ROM, to the best of our knowledge, this is the first study to analyze detailed active ROM recovery after arthroscopically assisted CC ligament reconstruction every 3 months. The recovery of active shoulder ROM was already noticeable 3 months after surgery despite the restriction of active ROM exercises for AE and abduction within 90 degrees for 1 month. Almost full recovery of ROM was achieved 9 months after surgery, and movement-concomitant complications, such as implant irritation and impingement syndrome, did not occur. In particular, AE and ER recovered almost completely between 6 and 9 months. The progressive recovery in AE and ER is attributed to bone union because complete bone union decreases pain during movement. In addition, this early phase recovery can possibly be attributed to enhanced construct strength against superior loading and reduced invasion of soft tissues with arthroscopically assisted procedures [28]. Enhanced construct strength against superior loading may lead to decreased pain during shoulder motion, with less fracture micromovement. Minimal invasion of soft tissues may enable early recovery of muscle strength and decrease the risk of the development of tight adhesions around injured tissue. Mini-open arthroscopy and accurate reduction can provide proper clavicle movement compared with distorted reduction or malunion. Therefore, early phase recovery of active shoulder ROM was accomplished for these reasons.

Regarding the functional outcomes, this study showed satisfactory clinical outcomes, with mean JOA and UCLA scores of 98.0 ± 3.1 and 34.3 ± 1.0, respectively, 12 months after surgery. Malik et al. [23] reported that shoulder function scores after arthroscopically assisted CC ligament reconstruction ranged from 81.8 to 96.2, as assessed through the Constant–Murley score. For other fixation methods, the mean Constant–Murley score ranged from 85.3 to 88 in the TBW group [35,36], from 79.9 to 93.3 in the hook plate fixation group, and from 82.8 to 98.1 in the locking plate fixation group [25,37]. On the basis of these scores, arthroscopically assisted CC ligament reconstruction may be superior to other surgical procedures. In addition, functional scores for CC ligament stabilization were better compared with other methods 3 and 6 months after surgery in a previous study [38]. These findings may be associated with the aforementioned early phase recovery. The present method also led to relatively better ROM recovery and better clinical outcomes compared with similar methods and other fixation methods, suggesting that arthroscopically assisted CC ligament reconstruction can be an optimal treatment for lateral clavicle fractures accompanied by ligament injury.

In the radiological outcomes, bone union was achieved in all patients, and the mean union time was 5 months. In a previous study on the nonunion rate after this fracture surgery, Raval et al. [21] reported a nonunion rate of 18% for fractures with CC ligament injury if treated properly with plate fixation. In other studies, arthroscopic CC ligament reconstruction with a suture device had bone union rates of 70–100% [13,14,15,17,18,19,27,39] and union times of 3.5–8.4 months [15,17,27]. The discrepancy in these results can be attributed to differences in the fixation methods, including fixation points, implants, and reductions. Meanwhile, the union rates for the TBW procedure ranged from 52.6% to 95.6% [9,40,41,42]. Uittenbogaard et al. [43] reported that the highest revision rate (30%) was observed with TBW compared with other procedures. Malik et al. [26] found that hook plate fixation had a good union rate (96.4%), and the mean union time was 3 months. Anatomical locking plate fixation also had good union rates (96.9%). Zhang et al. [25] demonstrated that anatomical locking plate fixation and hook plate fixation had almost the same union time. From these studies, plate fixation appears to be the best treatment regarding bone union rates and time to union. However, most articles in previous systematic reviews did not classify the fractures in accordance with the modified Neer type IIA and IIB categories. Type IIA fractures have comparatively larger lateral fragments of sufficient size for plate fixation compared with Type IIB fractures. This difference may explain the good bone union rates with plate fixation in the aforementioned articles. In the present study, the bone union rate was 100%, which may have resulted from the accurate bone tunnel placement based on the presurgical measurement and anatomical reduction with the mini-open technique. Otherwise, bone union time was later in this study compared to plate fixation methods. The late healing may have been triggered by the flexible fixation with the suture button device, which causes micromovement of the fracture site and leads to a longer time to achieve complete union. Slight superior displacement in CC distance and implant distance and bone hole dilations in the superior coracoid and inferior clavicle were also found with our method. However, there were no significant differences 3 months after surgery. There was also no implant migration and peri-implant fracture in the coracoid and clavicle. These findings indicate that sufficient superior stability was achieved during bone union using our method, and implant micromovement decreased after bone union was achieved. Hence, these changes did not appear to be critical events. Moreover, the low rate of critical complications indicates correct bone hole positioning, which was estimated before surgery. Accuracy can be achieved using arthroscopy, which results in a low risk of cut-out or migration of the bone surface.

No complications were observed with our procedure. In a previous review, the complication rate for CC stabilization of lateral clavicle fractures with CC ligament injury was 4.3%, which included one contracture and one loss of fixation [44]. Furthermore, the rate of major complications was 1.0%, including nonunion, implant failure, peri-implant fracture, deep infection, and coracoid and acromial fractures. The rate of minor complications was 3.3%, including delayed union, hardware problems, subacromial osteolysis, clavicular erosion, peri-anchor problems, loss of reduction, AC joint arthrosis, pain, and superficial infection. In addition, the predominant major complication was nonunion, and the predominant minor complication was hardware problems associated with CC stabilization. For other procedures, the complication rate for hook plate fixation was 46.5%, and the associated major and minor complication rates were 4.5% and 42%, respectively. In comparison, the complication rate for locking plate fixation was 25.7%, and the associated major and minor complication rates were 1.9% and 23.8%, respectively [44]. These findings indicate that complication rates were lower with CC stabilization compared with plate fixation. There were no implant-related complications with our method. This low complication rate can be attributed to the arthroscopically assisted technique, implant selection, anatomical reduction with the mini-open technique, and thorough fascia wrapping of the implants. Arthroscopically assisted techniques enable direct visualization of where to set the implant. The coracoid base has a complicated shape, and it is difficult to identify where to set the implant with imaging alone; it is easier to find the center of the coracoid base with arthroscopy. Without setting the implant center, there is a higher risk of a cut-out of the coracoid process or bone migration, which leads to loss of reduction. In addition, creating the smallest possible bone holes with Dog Bone drilling prevents implant migration and peri-implant fractures; thus, there was no loss of reduction in the present study. Mini-open reduction, arthroscopy surgery, and thorough fascia wrapping can prevent implant irritation. Less invasive treatment with arthroscopically assisted procedures prevents soft tissue adhesions, which lead to contracture. With these advantages, we believe that the implant choice and procedure described in this study are less likely to cause the abovementioned complications.

Our study has several limitations. First, this study was not a randomized controlled study but a retrospective observational study. Therefore, it is difficult to compare our technique with other surgical techniques. Second, the number of cases may be too small to show a significant difference compared with other procedures owing to the low rates of implant failure, delayed union, nonunion, and late recovery of functional outcomes. Third, the short follow-up time was a limitation. There is a possible risk of the development of superior clavicle dislocation or bone hole dilation. However, the follow-up in this study was comparable to that in the majority of other studies. Fourth, there is a risk of measurement errors with X-rays due to the potential for changes in measurement with angle differences. To minimize this risk, CT scans may be the best method for scaling items, but it is impractical due to associated costs, radiation risks, and ethical concerns. Therefore, we used two measurements to ensure consistency.

In the present study, our method achieved acceptable ROM recovery at the 9-month follow-up and excellent recovery at 1 year; excellent UCLA and JOA scores; excellent VAS scores for pain at rest, at night, and during motion; and excellent VAS scores for satisfaction and bone union in all patients, with no complications. The major advantages of the method compared with other implant fixation methods, such as TBW, hook plate fixation, locking plate fixation, and similar methods, are the lower implant-related complication rates that result from the mini-open arthroscopic technique and accurate reduction, implant selection, and thorough wrapping using fascia. These methods avoided peri-implant fracture and implant migration. Furthermore, there was no loss of reduction or implant irritation. Our method reduced the need for a second surgery to remove the implant and led to complete bone union. Hence, mini-open reduction and arthroscopically assisted CC stabilization using an AC Dog Bone Button device as a suspension system can be considered a favorable procedure for lateral clavicle fractures accompanied by CC ligament injury.

## 5. Conclusions

This study reports the results of arthroscopically assisted CC stabilization using the Dog Bone Button device for lateral clavicle fractures accompanied by CC ligament injury. CC stabilization can achieve early phase ROM recovery, satisfactory outcomes, 100% bone union rates, and 0% complication rates. This study suggests the possibility of the procedure to become the optimal treatment for lateral clavicle fractures accompanied by CC ligament injury.

## Figures and Tables

**Table 1:** Summary of the patients’ characteristics and final outcomes.

								Final ROM				
Patient	Age	Sex	Type of Injury	JOA-S	UCLA-S	VAS-P	VAS-S	AE	ER	IR	Bone Union (m)	COMPLs
1	50	M	fall	94.5	33	0	85	170	80	Th7	3	no
2	24	M	traffic accident	100	35	0	90	180	70	Th10	7	no
3	56	F	fall	100	35	0	95	180	80	Th4	5	no
4	44	M	bicycle accident	100	35	0	100	170	70	Th7	5	no
5	48	M	bicycle accident	92	33	2	90	130	50	L5	3	no
6	58	M	bicycle accident	98	35	0	90	180	80	Th4	7	no

F: female; M: male; JOA-S: Japanese Orthopedic Association score; UCLA-S: University of California Los Angeles score; VAS-P: visual analog scale score-pain; VAS-S: visual analog scale score-satisfaction; m: months; ROM: range of motion; AE: anterior elevation; ER: external rotation; IR: internal rotation; COMPLs: complications.

**Table 2:** CC distances and Dog Bone distances.

	Coracoclaviclar Distance (mm)					Dog Bone Distance (mm)				
Patient	1 w	3 m	6 m	9 m	12 m	1 w	3 m	6 m	9 m	12 m
1	8.0	7.3	8.3	8.0	8.6	33.9	37.5	36.3	36.7	37.0
2	4.4	4.70	5.4	5.8	6.3	32.1	30.1	31.4	30.9	32.3
3	4.4	4.8	5.0	5.2	4.9	25.3	27.2	27.2	27.7	28.7
4	10.0	11.4	12.3	*	13.3	48.5	50.8	53.2	*	51.9
5	5.4	8.0	8.7	7.7	8.3	42.7	42.2	42.1	42.3	44.1
6	6.6	8.9	9.5	10.1	10.3	43.7	45.1	45.9	46.1	45.7
mean	6.5 ± 2.0	7.5 ± 2.5	8.2 ± 2.7	73 ± 1.9	8.6 ± 3.0	37.7 ± 8.7	38.8 ± 9.0	39.3 ± 9.6	36.7 ± 7.7	40.0 ± 8.8

w: week, m: months; * missing data.

**Table 3:** Bone hole sizes in the superior coracoid and inferior clavicle.

Bone Hole Size (mm)										
	Superior Coracoid					Inferior Clavicle				
Patient	1 w	3 m	6 m	9 m	12 m	1 w	3 m	6 m	9 m	12 m
1	2.4	4.1	4.5	5.5	5.8	2.4	4.2	4.6	6.0	6.1
2	2.4	2.8	3.0	3.2	4.6	2.4	4.5	5.6	4.8	7.0
3	2.4	3.8	3.9	4.0	4.2	2.4	5.8	5.7	5.5	5.8
4	2.4	3.0	3.5	*	5.0	2.4	3.0	3.5	*	5.0
5	2.4	4.3	4.6	4.6	5.0	2.4	4.1	3.8	4.4	4.4
6	2.4	4.5	5.5	5.1	5.6	2.4	4.5	6.3	6.4	5.6
mean	2.4	3.7 ± 0.7	4.2 ± 0.9	4.9 ± 0.9 ^a^	5.0 ± 0.6 ^a^	2.4	4.3 ± 0.9	4.9 ± 1.1	5.4 ± 0.8	5.6 ± 0.9 ^a^

w: week, m: months; ^a^ *p* < 0.05 versus 1 w; * missing data.

## Data Availability

Data are contained within the article.
